# Supplementary material for: Modulation of cAMP metabolism for CFTR potentiation in human airway epithelial cells
Source: Sci Rep. 2021 Jan 13;11:904. doi: 10.1038/s41598-020-79555-w (PMC7807051; doi:10.1038/s41598-020-79555-w)
Supplement: Supplementary file 1 — Supplementary Figures. [file 41598_2020_79555_MOESM1_ESM.docx]

**Modulation of cAMP metabolism for CFTR potentiation in human airway epithelial cells**

**Jenny P. Nguyen**^1^**, Matthew Bianca**^1^**, Ryan D. Huff**^2^**, Nicholas Tiessen**^1^**, Mark D. Inman**^1^**, Jeremy A. Hirota**^1-4,*^

^1^Firestone Institute for Respiratory Health – Division of Respirology, Department of Medicine, McMaster University, Hamilton, ON, L8N 4A6, Canada
^2^Division of Respiratory Medicine, Department of Medicine, University of British Columbia, Vancouver, BC, V6H 3Z6, Canada

^3^McMaster Immunology Research Centre, McMaster University, Hamilton, ON, L8S 4K1, Canada

^4^Department of Biology, University of Waterloo, Waterloo, ON, N2L 3G1, Canada

^*^Corresponding Author

**Corresponding Author:**Jeremy A. Hirota, PhD
Firestone Institute for Respiratory Health – Division of Respirology, Department of Medicine, McMaster University, Hamilton, ON, L8N 4A6, Canada
Phone: 905-518-0745
Email: hirotaja@mcmaster.ca

**Supplementary Information**

**Supplementary Figures**

**Supplementary Figure 1: Concentration-response analysis of compounds with ABCC4 inhibition properties on extracellular cAMP from human airway epithelial cells.** Human airway epithelial (HBEC6-KT) cells were pre-treated with IBMX (20 μM), exposed to (**a**) MK-571, (**b**) Ceefourin-1, or DMSO vehicle control, and then treated with forskolin (10 μM). Cell culture supernatants were assessed for cAMP levels 24h post-treatment. The half maximal inhibitory concentration (IC_50_) values of MK-571 and Ceefourin-1 were found to be 0.2 μM and 4.8 μM respectively. Each concentration-response curve was normalized to positive control (IBMX + forskolin) with data presented as means ± standard deviations (n=4, MK-571; n=5, Ceefourin-1).

**Supplementary Figure 2: Consequences of non-specific PDE inhibitor IBMX on CFTR function using receptor-independent (forskolin) and receptor-dependent (isoproterenol) cAMP inducers.** Calu-3 cells were stimulated with IBMX (100 µM) in the presence of cAMP inducers forskolin (FSK) and isoproterenol (ISO). Max peaks obtained after the addition of the cAMP-elevating agent from the (**a** and **g**) time course analysis was used to generate the (**b** and **h**) concentration-response curves. Analysis of the concentration-response curves were performed for (**c** and **i**) AUC, (**d** and **j**) EC_50,_ (**e** and **k**) E_max_, and (**f** and **l**) Baseline. The time course and concentration-response curve were normalized to baseline over DMSO vehicle control. All data presented as ± standard deviations (n=5). **P≤*0.05; ***P≤*0.01

**Supplementary Figure 3: Consequences of pharmacological interventions of CFTR modulator VX-770, ABCC4 inhibition, and PDE-4 inhibition on CFTR activity using a receptor-independent cAMP inducer.** Primary human airway epithelial cells from two donors were treated with combinations of VX-770 (1 µM), MK-571 (1.8 µM), and Roflumilast (RF – 1 µM) in the presence of cAMP inducer forskolin (10 µM). (**a** and **e**) Transepithelial electrical resistance (TEER) measurements were taken for each donor (n=24 and 12, respectively). (**b** and **f**) A time course analysis was generated and analyzed for (**c** and **g**) AUC and (**d** and **h**) Max peak (n=6-18). All data presented as ± standard deviations. **P≤*0.05; ***P≤*0.001; *****P≤*0.0001

**Supplementary Figure 4:** **Full western blot images used in Fig. 2.** (**a**) ABCC4 and (**b**) CFTR blots. (**c**) ABCC4 and (**d**) CFTR total protein stain blots.

**Supplementary Figure 5: Experimental time course analysis for experiments in Fig. 3-7.** (**a**) cAMP-elevating agents forskolin (FSK) and isoproterenol (ISO) alone or with (**b**) CFTR modulator VX-770 (1 µM), (**c**) ABCC4 inhibitor MK-571 (1.8 µM) or Ceefourin-1 (4.8 µM), (**d**) PDE-4 inhibitors Roflumilast (1 µM) or Rolipram (10 µM), or a combination of ABCC4 inhibitor MK-571 or PDE-4 inhibitor Roflumilast with VX-770 treatment.
